# Repair technique for a rare partial anomalous pulmonary venous return associated with retroaortic innominate vein

**DOI:** 10.1186/s13019-021-01457-3

**Published:** 2021-04-15

**Authors:** Hanna Jung, Joon Yong Cho, Youngok Lee

**Affiliations:** Department of Thoracic and Cardiovascular Surgery, Kyungpook National University Hospital, Kyungpook National University School of Medicine, 41944, 130 Dongdeok-ro, Jung-gu, Daegu, Republic of Korea

**Keywords:** Congenital heart disease, Partial anomalous pulmonary venous return, Retroaortic innominate vein

## Abstract

**Background:**

Retroaortic innominate vein (RIV) is a rare vascular abnormality. Although RIV itself is asymptomatic, its presence in patients with partial anomalous pulmonary venous return (PAPVR) to the superior vena cava (SVC) is surgically challenging because a simple Warden procedure is impossible.

**Case presentation:**

A 16-year-old girl was diagnosed with tetralogy of Fallot, secundum, and sinus venosus atrial septal defect (ASD) at birth. She underwent total correction of tetralogy of Fallot and ASD closure at the age of 14-months. However, the diagnosis of PAPVR was missed. At the age of 16, she developed dyspnea on exercise. Echocardiography demonstrated severe pulmonary regurgitation, mild tricuspid regurgitation, and D-shaped left ventricle with paradoxical septal motion along with RIV and sinus venous ASD. Computed tomography confirmed RIV and PAPVR. Systemic and pulmonary venous blood pathways were separated by bovine pericardial patch, and pulmonary valve replacement was performed. Postoperative echocardiography demonstrated improvement of D-shaped left ventricle and laminar flow through the SVC and pulmonary veins. Postoperative computed tomography showed a well-reconstructed SVC and pulmonary venous pathway without stenosis. After an uneventful postoperative course, patient was discharged.

**Conclusions:**

PAPVR in patients with RIV may be surgically challenging to repair. We report the first case of successfully repaired PAPVR associated with RIV.

## Background

Retroaortic innominate vein (RIV) is a rare vascular anomaly, usually associated with tetralogy of Fallot (TOF), pulmonary artery hypoplasia, and right aortic arch [1]. In partial anomalous pulmonary venous return (PAPVR), some pulmonary veins (PVs) drain either into the systemic venous circulation or directly into the right atrium instead of the left atrium (LA). This causes a physiologic left-to-right shunt that can have various presentations [2, 3]. Although RIV does not cause symptoms by itself, its presence in patients with PAPVR with drainage into the superior vena cava (SVC) is surgically challenging because a simple Warden procedure is impossible. Here, we present a successfully repaired PAPVR associated with RIV.

## Case presentation

**Fig. 1:**
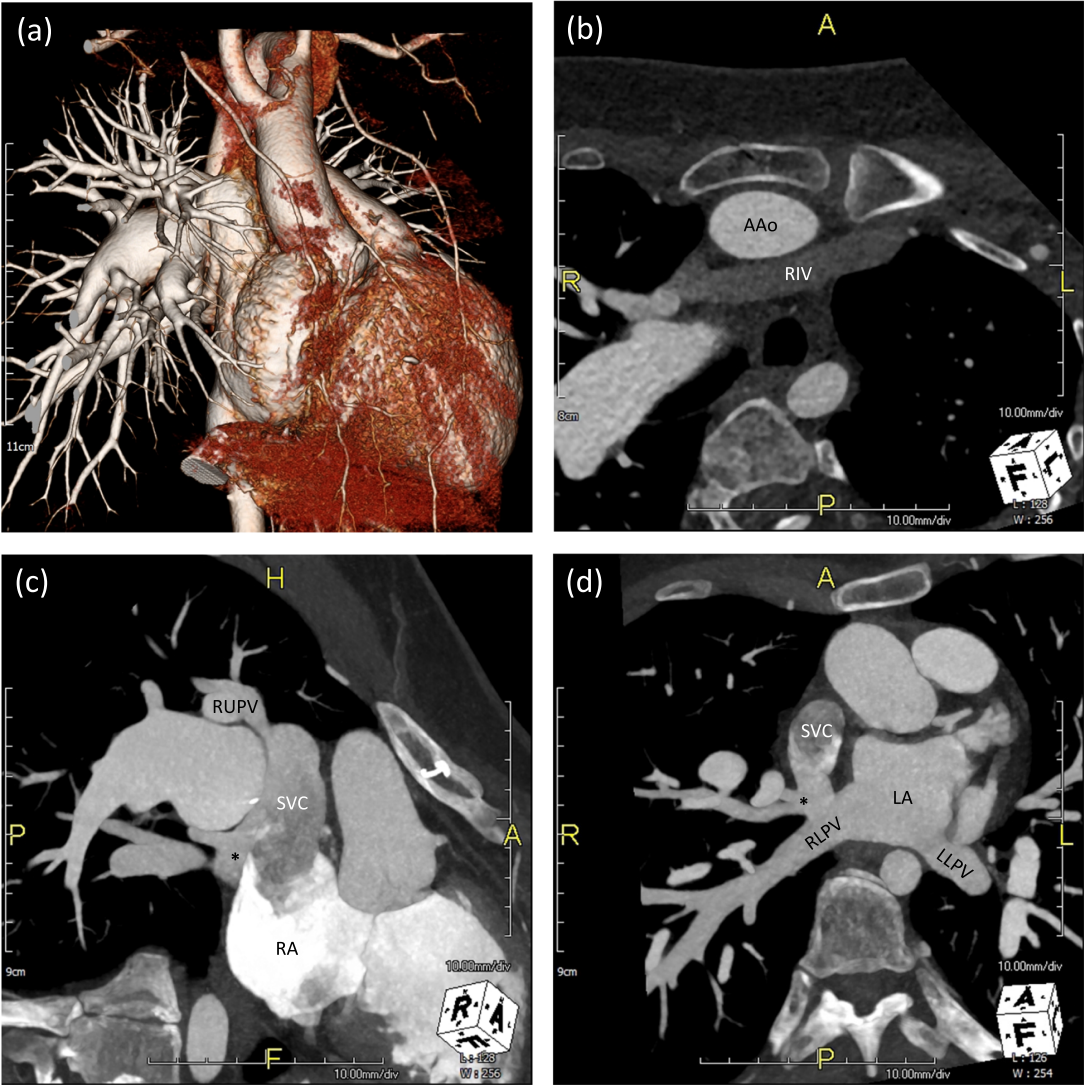
Preoperative chest computed tomography with (**a**) 3-dimensional reconstruction. Computed tomography demonstrating (**b**) RIV, (**c**) anomalous connection of RUPV and RMPV (asterisk) to SVC, and (**d**) dual connection of RMPV (asterisk) to SVC and LA. AAo, ascending aorta; RIV, retroaortic innominate vein; RUPV, right upper pulmonary vein; RMPV (asterisk), right middle pulmonary vein; SVC, superior vena cava; RA, right atrium; LA, left atrium; RLPV, right lower pulmonary vein, LLPV, left lower pulmonary vein

A 16-year-old girl was diagnosed with TOF, secundum, and sinus venosus atrial septal defect (ASD) at birth. Additionally, she had Turner syndrome [46, X, del(X)(p11.2)] and a horseshoe kidney. She underwent total correction of TOF and ASD closure at the age of 14-months, including patch closure of the subarterial ventricular septal defect, direct closure of the ASD, resection of the infundibular muscle, and transannular patch extending to the main pulmonary artery. However, the diagnosis of PAPVR was missed. She was doing well at her annual outpatient clinic follow-up. At the age of 16, she developed dyspnea on exercise. Echocardiography demonstrated severe pulmonary regurgitation, mild tricuspid regurgitation, and D-shaped left ventricle (LV) with paradoxical septal motion along with RIV and sinus venous ASD. Computed tomography also confirmed RIV and PAPVR (Fig. 1). The right upper pulmonary vein (RUPV) drained into the SVC and the right middle pulmonary vein (RMPV) showed dual drainage into the SVC and LA. Surgical repair of PAPVR, sinus venous ASD, and pulmonary regurgitation was planned.

**Fig. 2:**
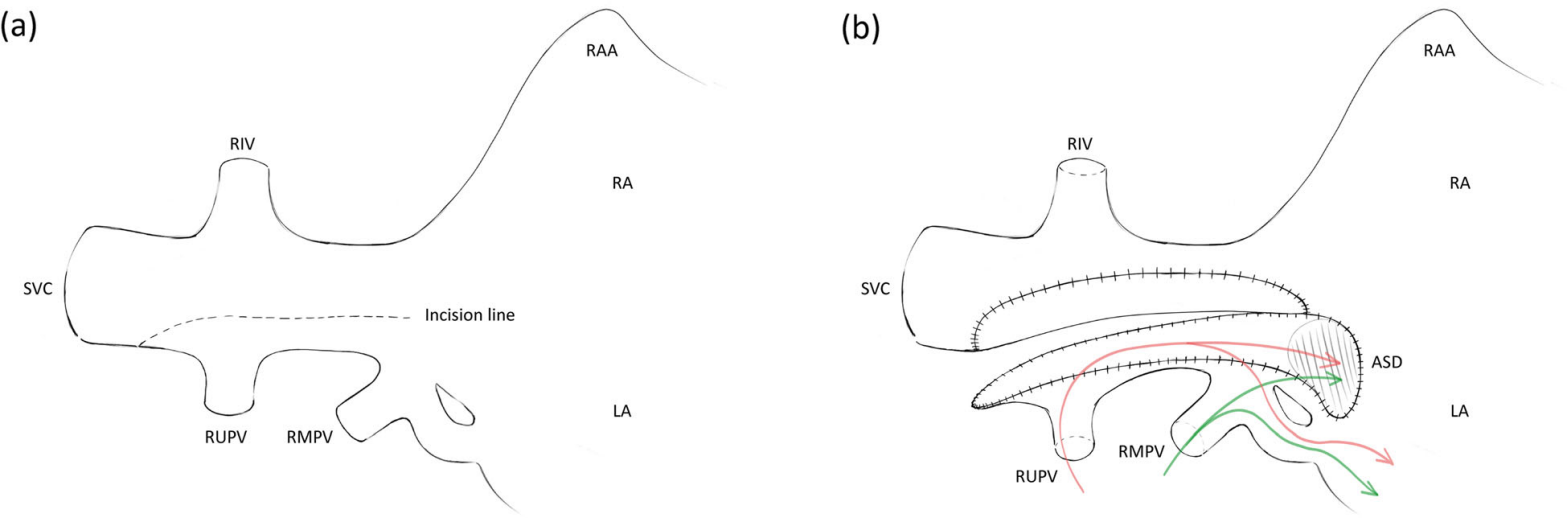
Image illustration of repair technique. (**a**) Lateral wall of SVC was incised from just above the junction of anomalous connection of RUPV to just below the junction of anomalous connection of RMPV. (**b**) Systemic and pulmonary venous blood pathways were separated by bovine pericardial patch. Creating dual pulmonary venous blood flow; RUPV and RMPV drained directly or through intra-atrial tunnel and sinus venous ASD into LA. SVC, superior vena cava; RIV, retroaortic innominate vein; RUPV, right upper pulmonary vein; RMPV (asterisk), right middle pulmonary vein; RAA, right atrium appendage RA, right atrium; LA, left atrium; ASD, atrial septal defect

**Fig. 3:**
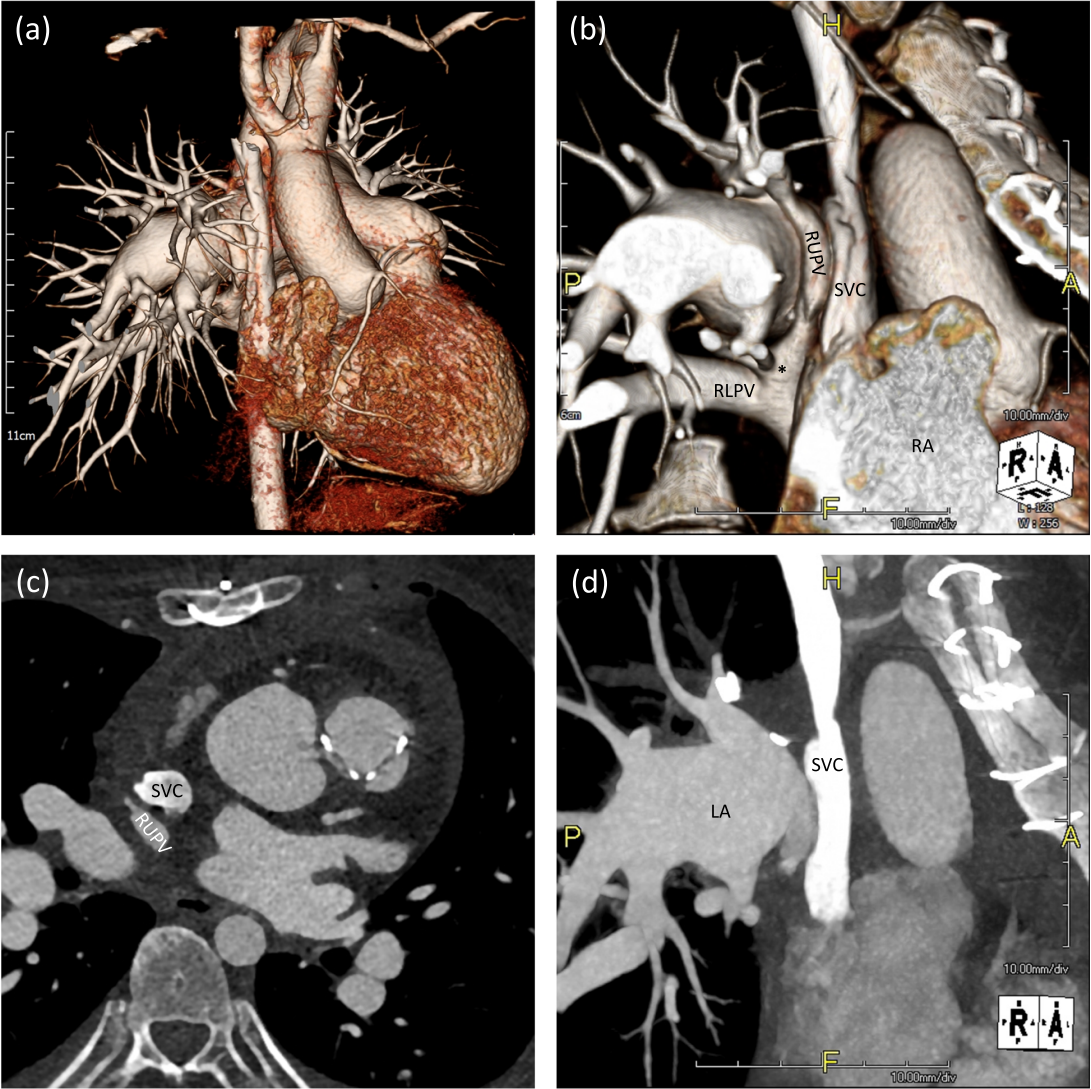
Postoperative chest computed tomography with (**a**) 3-dimensional reconstruction and (**b**) well-constructed SVC and pulmonary veins. Computed tomography demonstrating (**c**) RUPV and (**d**) LA separated from SVC. RUPV, right upper pulmonary vein; RMPV (asterisk), right middle pulmonary vein; RLPV, right lower pulmonary vein; SVC, superior vena cava; RA, right atrium; LA, left atrium

Redo-median sternotomy and bicaval venous cannulation were performed. SVC cannulation was performed with a 20 Fr angle catheter at the utmost distal SVC above the anomalous PV and RIV. Under mild hypothermia cardiopulmonary bypass and after pharmacological cardiac arrest, the lateral wall of the SVC was incised from just above the junction of the anomalous RUPV to just below the junction of anomalous RMPV (Fig. 2a). Systemic and pulmonary venous blood pathways were separated by bovine pericardial patch. Consequently, dual pulmonary venous blood flow was created; RUPV and RMPV drained directly or through the intra-atrial tunnel and sinus venous ASD into the LA (Fig. 2b). Pulmonary valve replacement was performed with a 25 mm Trifecta aortic bioprosthesis valve (St. Jude Medical, Inc., USA). The duration of bypass and aortic cross-clamp was 125 and 96 min, respectively. After an uneventful postoperative course, patient was discharged on postoperative day 6. Postoperative echocardiography demonstrated improvement of D-shaped LV and laminar flow through the SVC and PVs. Postoperative computed tomography showed a well-reconstructed SVC and pulmonary venous pathway without stenosis (Fig. 3).

## Discussion

The exact embryogenesis of RIV remains unknown. The dominant hypothesis is that RIV represents the persistent inferior transverse capillary plexus between the anterior cardinal veins during embryonic development. The descent of the aortic arch and development of the pulmonary artery normally leads to the degeneration of the inferior transverse capillary plexus, explaining the common association of TOF, pulmonary artery hypoplasia, and arch anomaly with RIV [1, 4].

In PAPVR, some PVs drain either into the systemic venous circulation or directly into the right atrium, resulting in a physiologic left-to-right shunt. Patients with hemodynamically insignificant shunts are asymptomatic with absence of right heart failure and conservative management is recommended with close monitoring [5]. However, if a significant left-to-right shunt is left untreated, pulmonary artery hypertension can develop leading to pulmonary vascular occlusive disease, Eisenmenger’s syndrome, and biventricular heart failure [2, 3]. Since our patient had symptoms and right ventricular enlargement, PAPVR repair and pulmonary valve replacement were indicated.

Several procedures have been reported for the repair of PAPVR with high insertion of PVs into the SVC. The Warden procedure or its modifications are preferred, although they have many long-term complications including sinus node dysfunction, SVC or PV obstruction, and supraventricular arrhythmias [2, 3]. RIV is asymptomatic by itself, but RIV in patients with PAPVR to SVC is surgically challenging because a simple Warden procedure is impossible. These patients may experience obstruction of the SVC or innominate vein more commonly after the Warden procedure because anterior mobilization of the innominate vein during the procedure may cause RIV compression by the ascending aorta.

Since our patient was diagnosed with PAPVR at 16 years of age, she had relatively large-sized PVs compared with younger pediatric infants plus dual pulmonary venous blood flow, making it possible and/or easy to repair PAPVR. Due to late diagnosis of PAPVR she have been distressed with right ventricle enlargement and D-shaped LV, perhaps her PVs size might have been too small at the time of the first surgery, making PAPVR repair technically difficult with poor prognosis.

## Conclusion

RIV does not cause symptoms by itself, but its presence in patients with PAPVR may be surgically challenging to repair. We have described a repair technique for SVC and PV reconstruction in patients with PAPVR. Through our case, infants with complex PAPVR, delayed or staged repair of PAPVR could be preferrable till patient grows up to have proper sized PVs to repair.

## Data Availability

Not applicable.

## Abbreviations

RIV: Retroaortic innominate vein
PAPVR: Partial anomalous pulmonary venous return
TOF: Tetralogy of Fallot
PV: Pulmonary vein
LA: Left atrium
SVC: Superior vena cava
ASD: Atrial septal defect
LV: Left ventricle
RUPV: Right upper pulmonary vein
RMPV: Right middle pulmonary vein
